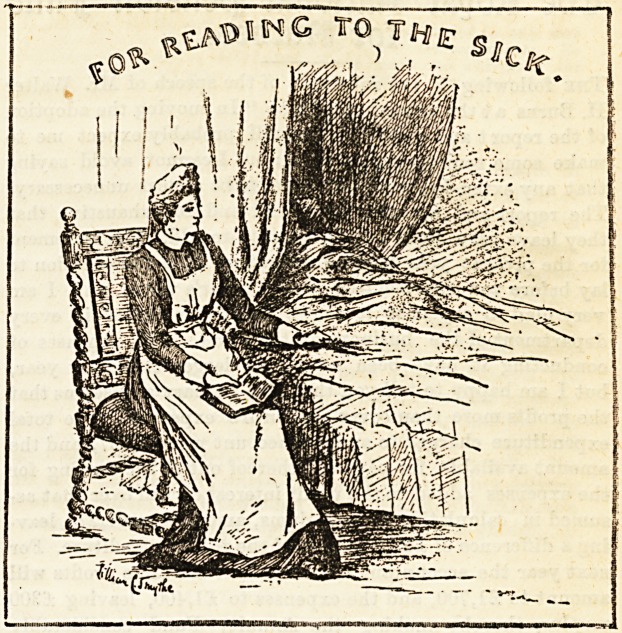# Extra Supplement—The Nursing Mirror

**Published:** 1891-03-21

**Authors:** 


### "The Hospital" Nursing Mirror.

BEING THE EXTRA NURSING SUPPLEMENT OF "THE HOSPITAL" NEWSPAPER.

Contributions for this Supplement should be addressed to the Editor, THE HOSPITAL, 140, Strand, London, W.C., and should have the word "Nursing" plainly written in left-hand top corner of the envelope.

### En Passant.

ADVANCE AMERICA.—The sisters of St. John the Divine, in their last annual report, innocently told a touching story of a wretchedly poor and dirty old man they tended. They washed him and put him on an old Howe's bed, upon which the patient remarked gratefully, "Oh, Sister, this would be Heaven if it were not for the pain!" The Sisters doubtless had no intention of advertising Howe's beds, but the amusing result is that the story is being strewn broadcast as an advertisement with the heading, "Howe's Beds are Heaven." After which it is needless to state that the company is of Yankee origin.

A NURSE AGED SIXTEEN.—There is no limit whatever to the stupidity of some Guardians. Those at Hackney have lately appointed as assistant night nurse in the male wards Beatrice Harriett Bedford, aged 16. How long will the public tolerate this sort of thing? Everyone knows what the duties of a nurse are, and everyone knows the special dangers which this girl has been appointed to face. Danger to the patients, danger to the nurse, and no reason whatever for thus breaking through the ordinary rules. It is no use to dwell on the subject, the facts speak for themselves. Would that the public would hear and unseat every one of these Guardians at the next election!

SHORT ITEMS.—With regard to the coming census, will all certificated nurses and midwives please use the term "certificated nurse" or "certificated midwife," instead of saying "trained," which is capable of a different interpretation.—We would point out to nurses that Messrs. Southall are now making a new medium towel, a specimen of which will be sent free on application to the Lady Manager, 17, Bull Street, Birmingham.—The South London Nursing Association, 178, Stamford Street, appeals for old linen.—Several of our queries lately have remained unanswered. May we hint that answers, even on post-cards, would be most acceptable, and that in this way nurses can help one another?—Salford Royal Hospital Committee has decided to start a private nursing institution.—The Society of Volunteer Nurses of the Sick and Wounded in War lately held their annual inspection at Berlin, in the presence of the Empress.

THE SPIRIT OF CHARITY.—And now one word about the total absence of the Christian spirit of love which seems to mark all charitable meetings. At nearly every gathering in aid of a hospital or institute at which we have been lately, those on the platform have tried to gather favour by abusing similar work in others. Of course, General Booth comes in for most of the abuse, but after all he has got the money he wanted, and no snarling or carping will make it change into other pockets. Then at a district nursing meeting one speaker made insinuations about the money wasted in building hospitals so large that when finished the wards were kept closed for want of funds. Then the East London Nursing Society utterly ignored the Haggerston and Hoxton Nursing Association, and spoke as though they alone did any nursing in the East-end. General Booth is constantly taken to task for this fault, and yet his vilifiers imitate him. "How these charities love one another!" scoffed a listener at one of these annual meetings, and then shrugged his shoulders and went away. Shall we seek in vain for charity amongst the charities?

HAGGERSTON AND HOXTON.—The East London Nursing Society, in the beginning of last week, stated that it longed to possess the whole of the East-end, but in 21 years it has only gained 27 parishes, and other equally good societies are in the field. At the end of the week the first annual meeting of the Haggerston and Hoxton District Nursing Association was held at Lord Meath's, Lord William Cecil in the chair, and Lady Salisbury amongst the audience. The association is affiliated with the Queen Victoria Jubilee Institute, and has its head-quarters in Nichols Square, Haggerston; already there are three nurses at work, and their services are very highly appreciated by the clergy, the doctors, and the people. The association owes its start chiefly to the energies of Miss L. D. Sparkes, Mrs. Franks, and Miss Alice Goodban, and we heartily congratulate these ladies on the quiet and efficient way in which they have organised a most useful institution. Lord Stamford, the Rev. S. J. Stone (author of "The Church's One Foundation"), and Dr. Hewitt Oliver pleaded eloquently at the meeting for further subscribers.

AN ANCIENT REMEDY.—Really it seems there is no reason why Miss Wilson and others should get indignant with union infirmary nurses, because they beat the sick old paupers under their care. In the ledger of a Huntingdonshire village, under date 1711, we find the following: "Pd. Thomas Hawkins for whypping 2 people yt had small-pox, /8'." Therefore, custom decrees that there may be something remedial or right in this treatment of the sick and dying. Perhaps that is why an enlightened member of the Richmond Board, when the case of a nurse dexterous with the rod was before him, said he thought that the only way to manage these people was by such means. "These people" stands for sick paupers, and we have a sort of a notion that to a pauper pain and death are as fearsome as to a millionaire. Some of the stories sent us are so shocking we could paraphrase Shylock, and cry, "Hath not a pauper hands, organs, dimensions, senses, affections, passions?" A notable case of workhouse discipline came before a London magistrate last week, in which a man for speaking at meals was put on bread and water, and given a punishment allowance of oakum to pick.

EAST LONDON NURSING SOCIETY.—The annual meeting of this society was held last week in the beautiful rooms of Lowther Lodge. The Princess Christian was present, and the Bishop of Bedford and Mrs. Frederick Beer and Miss Lowther—both the last well-known in East-end nursing circles. Lord Cranbrook took the chair, and stated that the Society looked forward to getting possession of all the East-end of London, or at least 100 parishes; at present it had 27 parishes on its hands. The Rector of Stepney gave some notion of what these parishes are. He said he had 22,000 people in his parish, whereas Canterbury, with a population of 21,000, had a cathedral and 15 churches and all their staff of clergy. The Rector of Stepney thought £50 could not possibly be better spent than in maintaining one of the nurses of the Society, and as there was a large and fashionable audience present we hope his words fell on fruitful ground, and that more nurses will shortly be added to the staff. The Hon. Mrs. Stuart Wortley spoke in behalf of the Society with which she had been so long connected, and a collection made by the nurses concluded the meeting. The report, which was taken as read, tells of 83,593 visits paid; the staff consists of four Matrons and 27 nurses. There is a deficit of £127 on the year's working, and the Society needs to be more widely known that its good work may receive more support.

### Lectures on Surgical Ward Work and Nursing.

By Alexander Miles, M.B. (Edin.), C.M., F.R.C.S.E.

### Lecture XVII.—PREPARATION OF PATIENT FOR OPERATION.

This includes (1) the preparation of the patient himself for the anæsthetic, and (2) the preparation of the part to be operated upon.

(1) The Preparation of Patients for Anæsthetic.—In the case of a patient who is in the habit of leading an active life out-of-doors, it is always wise before subjecting him to a serious surgical operation to keep him in hospital for a few days, that he may get accustomed to his altered conditions and his new surroundings. The last day or more of this probation may be spent entirely in bed. He should also be specially dieted for the same period, fish, chicken, milk-puddings, and similar light nourishing diet being indicated. Special attention must be paid to his excretory functions, notably his alimentary track. The bowels should move regularly and naturally, and if this does not occur spontaneously, it must be secured by administering suitable medicines under the doctor's orders. On the evening immediately preceding operation the patient should have a large dose of castor oil (say a tablespoonful and a half for an adult, or a dessert spoonful for a child), and about four o'clock the next morning the night nurse will give a large enema of soap and water, to ensure complete evacuation of the bowels. Supposing the operating hour to be noon, about 6 a.m. the patient should have a light breakfast consisting of a cup of tea and some plain dry toast, and he should have *nothing* after this. In the case of children and not unfrequently in adults, it is necessary to watch that food is not obtained surreptitiously as it is difficult to persuade them that this prolonged fast is necessary. In very old and feeble patients of course certain allowances must be made. Carefully regulating the bowels by small doses of cascara for a few days will dispense with the necessity for a large purge, and a small plain enema in the morning will suffice. Such patients, too, may have beef tea, Valentine's meat juice, brandy and similar fluid supports, but nothing solid. The necessity for these precautions being carried out to the letter will be evident after what I have said of the dangers of sickness and vomiting during anæsthesia.

(2) The Preparation of the Part to be Operated on.—On the evening before operation the skin, if unbroken, should be washed with soap and water to which some soda has been added, to dissolve the superficial epithelium, and to remove oil and grease. It is then covered over with a carbolised towel (1 in 20), kept moist by a layer of macintosh, and renewed at morning visit. This renders the skin antiseptic, and by softening the epithelium enables it, and its contained germs, to be easily removed before beginning the operation. It is often an advantage to shave the part before applying the carbolised towel, but with nervous patients this may be left till after the anæsthetic has been given.

Taking the Patient to and from the Theatre.—When available a wheeled table is by far the best means of removing a patient from the ward to the operating theatre, and as this is also the operating table he can be quietly and comfortably arranged in the ward so that as soon as he arrives in the theatre the adminstration of the anæsthetic may be commenced, before ever he has had time to become alarmed by the preparations made for his much dreaded operation. This leads me to speak of the question of administering the anæsthetic in the ward, or ante-room, before taking the patient into the theatre. As a general rule this is not advisable. In transit between the two places any accident may happen, such as the patient fainting, or vomiting and choking on the vomited matter, and such an accident may escape notice, or the requisite means of treatment be unavailable, with the most serious results. I have heard a surgeon of long experience state that the only serious accidents he has seen in the administration of anæsthetics have occurred between the ward and the theatre. When a stretcher has to be used the patient should be carried as steadily as possible, the bearers "breaking the step," to avoid the uncomfortable swinging which results when they keep in step. It is obvious that if repeated shifting of the patient from one thing to another is undesirable in taking him to the theatre it is much more so in removing him back to bed, when he is in a partly unconscious condition, suffering from shock, and very probably has some wound which will be injured by moving. It is here that the wheeled table is particularly useful. Never under any circumstances prop a patient up in the sitting posture immediately after an operation. The risk is that the weakened circulation will not be sufficiently strong to carry blood to the brain, with the result that the patient will faint, and even a more dreadful result may follow. This danger always exists in patients under anæsthetics, and you must be ever on your guard lest it occur to you. It is a common thing to see a child semi-anæsthetised lifted in a nurse's arms and carried from the theatre. If this is properly done it is quite a safe procedure, but in nine cases out of ten it is not properly done. The child is seized with one arm around its neck, and other behind its knees. The head hangs over the arm which supports it, while the limp body doubles up and hangs between the two arms. In this position, although the head is hanging, it is by no means the most dependent part of the body, as it should be. It is almost the highest part. The child's buttocks are its lowest parts, and the position is perhaps the very best that could be wished for allowing all the blood to gravitate into the abdomen and pelvis, so depleting the brain and causing fainting. I have seen an alarming accident caused in this way, and I warn you against it. If you will carry a semi-conscious child in your arms, make sure that his head is the most dependent part of his body.

The Nurse's Duties towards Patient during Operation.—It is an invariable rule that a nurse should accompany every female patient to the theatre, and remain with her throughout. It is comforting to the patient, and many of her fears are dispelled when she has by her a friendly nurse whom she has come to know and trust, rather than a host of strangers. The patient's dress should always be arranged, so far as is possible, before leaving the ward. For example, when the operation is in the region of the shoulder or chest, the night-shirt should be removed from these parts, and a blanket take its place till the operation is begun. In operations about the face and neck, especially in females, much discomfort is caused to the patient, and no less trouble to the nurse, by the hair getting soiled and matted with blood and lotion. This is very easily obviated by covering in the whole head with an india-rubber cap, such as ladies wear while bathing, and then this by a dipped towel. A female patient's hair should never be plaited and then coiled on the back of her head, because it becomes very painful when she has to lie for a long time resting on it. Other arrangements of the dress of patients to prevent chilling or undue exposure are obvious. When the patient is weak and collapsed, or very old, he should have hot bottles placed on the table, care being taken that they are so placed as not to interfere with the surgeon, and especially that they will not fall off the table on to his toes.

### Notice.

At St. Thomas's Hospital on the 24th inst., at five p.m., a presentation will be made to the Founder of the Pension Fund by the First Thousand members of the Fund. Sir Andrew Clark will preside. All nurses admitted.

### The Eastern Hospital Scandal.

ON March 12th Arthur Jay, recalled, deposed, in re-examination, that he did not complain to the Matron because he did not know she was the proper person to complain to, and he never saw her speak to any patient while he was there. By Mr. Hedley: During the whole time he was in bed in the hospital Dr. Collie never spoke to him once. Dr. Collie never felt his pulse, and the nurse took his temperature.

William Haynes, a lad of fifteen, working at a printer's, and living at 96, Chapman Road, Hackney Wick, deposed that he was a patient in the Eastern Hospital last autumn for ten weeks. How was the food? Bad. The bread was stale and sour, and he could not eat the fish, because it was so bad. The eggs were generally musty and bad. They never had any butter in the hospital. What they did have was very rank margarine. The potatoes were sent up half bad at times, cold, and in their skins. The meat supplied was mutton, always boiled and inferior. While he was in the hospital he was placed in a large bath several times with other patients—two and three others at the same time. He had been sent to other wards to borrow food, and had been told by the nurses that he was always begging.

Alexander William Grant bore evidence as to the bad food and deficient clothing; he was three weeks as a patient in the Wigram Ward before he saw Dr. Collie.

Robert Judkins, a Post-office official, said that his child had been to the hospital. When the boy, who was 3½ years old, returned home he could not eat. He was in a very weak state, and his hair was full of vermin. The child afterwards died of typhoid fever. Witness wrote to Dr. Collie about the child, but he replied that it had been sent to Winchmore Hill. Mr. Hedley: It appears that your son was at Winchmore Hill, and we are not inquiring into that institution. Witness: But his hair ought to have been cut, and it ought not to have been full of vermin. Mr. Hedley: That is so; but we are only inquiring into the Eastern Hospital.

William Anthony, a laundryman employed at the Eastern Hospital, said he had been at the hospital for fourteen years. The clothes supplied to the patients were very bad until Simpkins's letter appeared in the papers. He had seen good clothing destroyed. He had seen hundreds of yards of carpeting destroyed. Those things were burnt in the crematory at the hospital. He remembered Simpkins's letter, and since that there had been a difference in his work in the laundry. There had been an increase in his work. About the end of April or beginning of May, 1890, he saw Nurse Sara standing at the door of a ward crying. She told witness that she had twenty-seven patients in bed totally unable to help themselves, that the work was too great, and that she could get no assistance.

Sarah Clements, a laundress at the Salvation Army Rescue Home, Mare Street, Hackney, said she was laundry superintendent at the hospital from August, 1886, till last year. The clothing was, generally speaking, in bad condition. She had seen good clothing destroyed. A great change took place after Simpkins's letter appeared in the local paper. Witness heard Nurse Anderson complain that she was left without proper assistance. Nurse Anderson said her patients were dying off so, and she (the witness) said that it would be called manslaughter out of doors. That seemed to frighten Nurse Anderson. This conversation took place in the corridor outside the St. Veronica Ward. She had seen stinking fish brought into the hospital. It was accepted as good. The inquiry was adjourned.

---

ON February 18th the two new wards which have been added to the Heywood Cottage Hospital, Burslem, through the munificence of Mr. Matthew Dobson, who has given £1,000 towards this object, were opened with public ceremony. The new wards give accommodation for twelve more beds, raising the total number in the hospital to twenty-five, of which twenty-three are occupied. The hospital was opened in 1887, and has proved of much service to the poor of the neighbourhood.

### HELP IN SUFFERING.

A LIFE of suffering perhaps lies before me, how can I bear it? This question comes to the mind as day after day passes and we gain no relief from the pain which consumes us.

There is a common-sense answer to this, namely, try and make the best of it, and it is very sensible advice, for if we are always complaining we make ourselves worse, and wear out the patience of our friends and attendants. Yet it sounds very cold and unsympathising to the sufferer's ear. But let us grasp the idea and see what helps we can get to carry it out, for, indeed, there are many we little dream of. The kind word, the gentle touch, the interest which our case excites, are all soothing to the nerves for a time, but we find we want something more which no earthly help can give. Then we realise that it is God alone on whose loving breast we can throw ourselves. He knows how weak and feeble we are, and will not send us more than we can bear. Let us remember we are not alone in our agony, but united closely with our dear Lord, whose cross and passion borne for our sake will sanctify our affliction to us. Every pang we feel will purify our souls and bring us nearer to Him, so that at last we shall welcome the sufferings which make us more like Christ in patience and resignation. He came to do His Father's will on earth, and carry the punishment of the sins of all mankind. It was a fearful doom for a pure and sinless Being, yet He never drew back, only prayed that, if possible, "the cup might pass," and then was strengthened to drink it to the dregs. Christ went not up to glory, but first He suffered pain; He entered not into glory before He was crucified. We may take Him for our example, and ask in reverence that our cup of bitterness may pass, and if God be willing it will do so, but if He sees it is for our good to let the waters of affliction go over our head, we, too, shall receive heavenly support to say with true resignation, Thy will be done. Oh! what comfort to feel that, wherever our bed may be, we lie in the Lord's hands; that, however restless we feel, there is a place of repose in store for us, where Jesus has gone before. He, our elder Brother, learned obedience by the things which He suffered; we, then, will not despise God's chastening, nor faint when we are rebuked, for the Lord loveth whom He chasteneth, and scourgeth every son whom He receiveth.

Thou art our Pattern to the end of time,
Oh, Crucified! and perfect is Thy will,
The workers follow Thee in doing good,
The helpless think of Calvary, and are still.

### The Royal National Pension Fund for Nurses.

The following is the substance of the speech of Mr. Walter H. Burns at the annual meeting: "In moving the adoption of the report and accounts, you will probably expect me to make some remarks upon them; but I cannot avoid saying that any extended comments seem to be almost unnecessary. The report and accounts are so full and so exhaustive that they leave very little for a Chairman to add. The statement for the present year is the first that we have had occasion to lay before you representing a clear year's work, and I am very glad to say that it shows distinct progress in every department of the business of the Fund. The expenses of conducting it have been somewhat larger than last year, but I am happy to tell you that our Actuary advises us that the profits more than cover the entire expenses. The total expenditure charged to annuity account was £1,257, and the amount available for payment thereof out of the loading for the expenses added to the profit interest earned over that assumed in calculating the premiums, amounts to £1,279, leaving a difference of £22 in excess of the total expenditure. For next year the accountant estimates that the same profits will amount to £1,700, and the expenses to £1,400, leaving £300 surplus, thereby making the Annuity Fund considerably more than self-supporting. From the above vantage ground it is scarcely likely to recede. The preceding is, I am assured, an unusual experience for a new insurance company, and, while we have reason to congratulate ourselves upon the past, the outlook seems very brilliant for the future; and should our present rate of increase go on, which is about an average of one new nurse every day, before 20 years are over it is calculated that our invested fund, which is now nearly £100,000, will reach the neighbourhood of a million of money. The Benevolent Fund has now, as you will notice by the report, reached £10,000, and is certainly a noble memorial to the memory of our benefactor, Mr. Morgan. The fund has scarcely yet gone into active operation, but the organisation is completed, and probably at the end of the coming year, your Chairman will be able to give you some report of its working. It is with great pleasure that I have to announce to the Society that Lady Cadogan has consented to take the place of the lamented Lady Rosebery as a Patroness, and to aid in the distribution of the Benevolent Fund. During the year a new branch has been introduced into our business, and as it may give ground for some little misconception, I think it well for me to dwell more fully upon it. I allude to the Savings Bank Scheme, which was initiated for the purpose of assisting nurses in the payment of their premiums. The plan is to receive money at such time as the nurses can deposit it, and when it amounts to the sum of £10, to invest the same in the payment of the premiums upon their policies. The object of this was especially to enable private nurses, whose income is irregular and fluctuating, to make payments when they were able, and to suspend them for such period as was convenient. We found in practice that a good many nurses were deterred from taking out policies from the fear that they could not make their payments regularly, and might find their policies vitiated by no fault of their own. We think that the plan we have devised is one entirely safe for the Fund, and our Actuary, Mr. King, has so reported to us; but we also think that it will be a great boon to the nurses. The danger is that it should be misconstrued by them into a regular savings bank operative account, and against this construction I desire specially to warn them. The Council in no sense intended this when establishing it, nor will they receive deposits, subject to withdrawal, except upon such grounds as would justify them in assenting to the withdrawal of any payments under an ordinary policy. The object I cannot too strongly impress upon the nurses is to receive their money in advance of dues, so that they may be in a position to delay payment if their convenience requires later on; but not to be drawn out as an ordinary savings bank deposit. The Council have given a good deal of consideration during the year to the working out and elaboration of the affiliation schemes for other hospitals. A plan has been drafted by the Manager (to the hospitals and institutions), showing the best method for federating on behalf of their nursing staff. There is a counter-balancing advantage for any outlay to the institutions themselves, by the insurance of a permanence among their staff, of greater thrift and economy among the nurses, and the undoubted fact a sure protection for old age gives greater energy and better services during youth. I may mention that during the year assistance has been given to certain nurses by grateful friends and patients, and this is a form of benevolence which we think cannot be too earnestly commended to the public. There are many who benefit by the services of nurses, and who do not know how best to testify their gratitude in a permanently useful way. A policy, either on the returnable or non-returnable scheme, meets just such a want, and we are glad to see that there is a disposition on the part of the public to avail themselves of it. The Council have further matured a plan by which the existence of such a provision may be kept secret from the nurse herself, the insurance being made in a manner in which the name is withheld from public knowledge. This provides for cases where it is feared that knowledge of the possession of a policy might be injurious in some way to the beneficiary. I have been very dry in the details that I have given, because it seemed to me that it was better to lay before the public on this occasion the business portion of our institution more fully than hitherto. We have passed out of the tentative stage, and thanks to the assistance of the many hands and hearts that have fostered our scheme, and the patronage of the Prince and Princess of Wales, and the hearty and gracious endorsement they gave the nurses at Marlborough House, public attention is now fully awake to our existence, and nurses are coming in for insurance more rapidly than any of the Council for a moment anticipated. One result of the reception of the First Thousand nurses by the Prince and Princess of Wales at Marlborough House on the 4th July last is that her Majesty the Queen has been pleased to command that the Fund shall henceforth be called "The Royal National Pension Fund for Nurses." This spontaneous mark of the Queen's interest in and approval of the Fund is certainly most gratifying. I think it is an opportune moment to place before the nurses the additional facilities we have devised for them, and before the hospitals and institutions the opportunities we are affording them as well. I can only trust that the past may be an earnest of what the future is to be. If so, the work which our founder, Mr. Burdett, commenced single-handed, and which of late years has met with such efficient and disinterested co-operation from our other fellow-labourers, will grow until it becomes one of the principal benevolent institutions of London. I have the honour to move that the report, accounts, and balance-sheet be adopted."

### Princess Christian's Daughter.

Miss E. Durham, Farringford, Freshwater, Isle of Wight, acknowledges the following additional subscriptions towards a wedding present for Princess Louise of Schleswig Holstein to be given as a proof of the gratitude of nurses for the interest Princess Christian has ever taken in their progress. Subscriptions will be received until the end of March, and will be acknowledged in these pages. Between March 9th and 16th the following sums were received:—

Superintendent H. Stewart, "Royal Red Cross," 5s.; Superintendent Alice Bolton, 5s.; Sister Alice M. Farmborough, 2s. 6d.; Sister Margarett, 2s. 6d. *Nurses* (1s. each): Mary Stockwell, B.N.A., Mary Rodwell, E. Wall; Addie, Emma, and Rachel, B.N.A.; H. Elliott, F. Turner, M. Davis; S. J. Collier and M. Bryns, B.N.A.; M. C. Arnold, L. Mason; Ellen Simpson, A. Hinkley, and M. E. Smith, B.N.A.; Grace Freeman, Nurse Goodman, Nurse Mills, Nurse H. Arnold, Nurse Cutts, Nurse Bolton, and Rodgate (second subscription).

### Jottings from "Gib."

THE Colonial Hospital was bought from the Spanish by the English Government about two years ago, when it, for the first time in its existence, acquired a Matron, and, later, Sisters, but decided to retain the native nurses.

It fell to my share to receive an appointment as Sister. Of course the greatest difficulty was the Spanish language, and the next, working the ward, with two male native nurses, for twenty-five beds, and no ward maid. It is only fair to say these native nurses are, as a rule, kindly and pleasant in manner, obedient when my orders were fully understood, which was often a lengthy piece of business, owing to my very imperfect knowledge of Spanish and theirs of English.

Many of these native nurses, indeed most, are extremely dense, and in total ignorance of the rudiments of nursing. A coalheaver on Monday may be the new nurse offered as the only available person for Tuesday; experience sadly proved the bulk of them quite unsuitable for attending to the sick, and, it must be confessed, very few were teachably intelligent.

Never will it be forgotten what happened during my "off-duty" evening. The doctor ordered an injection of warm olive oil for a man seriously ill, meaning it to be given by the nurse on my return. The nurse accosted me as I came in with, "I made the patient drink the oil in spite of his objections," and in an aggrieved tone added, "Now he says he feels sick." This happened during my first few days amongst them. Such an experience is, to say the least, a shock to patient and Sister.

Frequent inquiry concerning the washing of patients is necessary. The average Englishman gives no trouble on this score, but when it is remembered that eight or nine different nationalities are often to be found in one ward, there are many who cling to the opinion that to wash a sick man is highly dangerous; and, indeed, so it would be if left to the tender mercies of the native nurse.

The changing of the bed linen used to be a remarkable performance. The first time I required the nurses to change an under-sheet, thinking they would be sure to need it explaining, I added, "But come, and I will show how it is to be done." "We know all about it, sister," and evidently wishful to prove it, while I went for the clean sheet, each seized a corner at the bottom of the bed, and by main force brought both sheet and patient to the floor, much to my consternation, and their expressed surprise.

Indeed, the material was painfully raw, repeated daily instruction most necessary, but they did improve to a certain extent, and at a very slow rate. The majority of the patients were sailors from the merchant vessels, representatives of many countries. Christmas Day saw a motley crew of convalescents around the dinner table, when the English roast beef and plum-pudding, ornamented by a miniature "Union Jack," were criticised in many tongues, and the ward decorations so kindly done by hospital friends caused much surprise and admiration.

Of course, many of the patients were Spaniards, and it is not always an easy thing for the English to avoid giving them offence, but when it is remembered that the Spaniard says "Gibraltar is temporarily in possession of the English," we "see ourselves as others see us." Even the way a sturdy Highlander marched down the ward caused annoyance, one man remarking in Spanish to his neighbour, "Look at him shaking his little petticoats with pride; he knows he has taken our Gibraltar."

The more remote historical event of the Spanish Armada was an unwelcome subject, but it was turned to good account on behalf of a Spanish soldier who was difficult to rouse after an anæsthetic. The happy idea struck some one to ask him, with an accompanying shake, if he had forgotten the Spanish Armada. To judge by the expression of his face, it was neither forgotten nor forgiven.

Hospital work, even at so short a distance from England as Gibraltar, in spite of our efforts on its behalf, was a very different thing from work at home, due to more causes than one.

### Everybody's Opinion.

*[Correspondence on all subjects is invited, but we cannot in any way be responsible for the opinions expressed by our correspondents. No communications can be entertained if the name and address of the correspondent is not given, or unless one side of the paper only be written on.]*

### NURSES' HOME, TRURO.

NURSE DENTON writes: I have read your paragraph in last week's HOSPITAL (February 14), under the title of "Truro Nurses' Home," and I should like to say a word in connection with the sentence with which the article closes. From this sentence it is clearly insinuated that the nurses are expected, and obliged to do, some "laundry work." Now, as I happen to have been a nurse engaged in the Truro Nurses' Home for some time, I can, I think, speak with some authority, and at all events knowledge, on the subject, and consequently I hasten to contradict the impression that your article conveys on this particular matter. It is absolutely untrue that the nurses are obliged or expected to take part in the performance of any laundry work. No doubt, if it amuses any nurse to spend some of her leisure time in the pursuit of knowledge as to laundry details she can do so, just as she might spend her time in the garden, or any other part of the domestic ménage.

### A NURSE'S HOURS.

"ONE IN THE RANKS" writes: Seeing this question of nurses' hours is again to the fore, may I venture a few remarks? What does our beloved pioneer of nurses, Miss Florence Nightingale, think of her would-be followers? She never grumbled about long hours; her whole life was just devoted to her work, the highest any woman can take up. Certainly we are over-tasked, but I firmly believe that the hardest workers are those who would fain keep their self-denying lives in the "dim distance." I am a certificated nurse; my hospital days were the happiest I have spent, and though often too fatigued in body to sleep, yet the feeling of usefulness and help to the suffering was enough in itself to keep me up. The long, really long hours (15½ in the 24), that I now take on duty in my capacity of private nurse, is far more tiring than hospital routine, and the friends of the patients generally fail to see why a nurse can possibly require rest and freedom. The pay is bad, very bad, but in the end it will right itself. Take Florence Nightingale for our password, and the Master for our example, and nurses will be helped on in their arduous duty.

### Presentations.

DR. J. E. CANE was, on Tuesday, presented with a plated-silver biscuit canister by the nurses of the Torbay Hospital as a mark of their appreciation for his kindness in giving them lectures for two winters. Dr. Cane is leaving the hospital to begin private practice in the neighbourhood of Torquay.

MISS EMILY STENDELL was greatly surprised and pleased to receive a very handsome afternoon tea service and a carved oak tray from the nursing staff of the Bristol Children's Hospital on resigning her appointment as Surgical Sister.

### Notes and Queries.

### Queries.

(44) *Macintosh.*—Where can I get thick drab macintosh? I find the white is generally kept.—*D. M.*

(45) *India-rubber Goods.*—Can air and water pillows be repaired?—*Economy.*

### Answers.

*For the Truth.*—We have long since given up contradicting such statements as those you enclose. If you do not like them there is an easy way of avoiding them—do not read the paper in question; we never do.

*Defense.*—You do not enclose name and address, and the comparison you make with the London Hospital is most unjust.

*A. G. B.*—The lines are good, but they do not scan correctly. We cannot insert these, but we should be glad to hear from you again.

(38) Babies under six months should not be fed after twelve p.m., nor before seven a.m.; over six months, the last feed should be at ten p.m. They then sleep well and thrive well.—*Nursie.* [This is very nice in theory, but in practice some babies won't stand it. We should like to hear from other nurses on the subject.—EDITOR.]

*M. M.*—The lunar month; thus, if a nurse arrives on a Friday she leaves the third Friday after.

*A Martyr.*—Cleanliness is the only possible precaution.

*Nurse M. B.*—There was no apology necessary; you shall hear all as soon as possible.

*Lodgings.*—The Temperance Hotel, Bridge Road, Blackfriars, is a suitable place for two nurses to stay at during a few days' visit to London.

*Scottish Women's Benefit Society.*—E. Royce is requested to send full address.

### Jarvy's Band.

"Dear, dear! What's the matter here?"

A sound of muffled weeping—weeping with a hopeless ring about it—fell upon my ear as I opened the door, and peered into the dim, quiet room.

"Jarvy!" I spoke again, "don't you know who it is?"

Then the wailing ceased; the quilt was pushed down from a small white face, and two drowned blue eyes looked out upon me from the bed in the corner.

"Oh, it be you, Nurse Grey! I heard the knocking, and I was afeerd it might be Miss Storkey; but I might ha' knowed it warn't, for she never knocks, she jest heaves herself into the room. I'm main glad its you, Miss, instead."

Miss Storkey is the district-visitor of our parish, and I, Bessie Grey, am the district-nurse. Evidently, comparisons are drawn between us of which I must not be over-vain.

"Miss Storkey is kind and thoughtful, Jarvy," I began, as I seated myself, and smoothed back the tangled curls from the tiny cripple's brow.

"Gran' doesn't think so," interrupted Jarvy; "and," went on he, a pitiful sob, belonging to the trouble about which I should presently hear, shaking him from time, to time, "she says Miss Storkey prys and spys round the poor man's 'ome; an' its quite true. When she comes in, she keeps on asking questions, while she looks in the cupboard, and lifts the lid off the pan Gran' has left bilin' on the fire, to peep inside. Then she says: 'Be you prepared for your death-bed?' and with that she heaves herself away again."

"Well, Jarvy, after that, I daren't offer to warm up some soup, eh?"

"Oh, Miss—nurse, I means—you be different, you be!" and Jarvy's thin feather-weight of a hand clutched at mine; "You be a sight for aching eyes, Gran' says."

"Come, now, Jarvy, don't talk nonsense. But I want to know the why of these tears; and then I must attend to that poor old back of yours."

"Well, it was in this way, Miss. You remember the band that I buyed?"

Jarvy, who had a devouring passion for music, had bought, with his savings, a cheap musical box, which was the crowning joy of the afflicted mite's existence. During the long hours when Gran', the bread-winner, was away, Jarvey's sufferings, however great, never refused to be soothed by his "band." And many a time had I cause to bless the toy, for I needed but to set it chiming, when I tended him, to see the lines of anguish smoothed out of the wee face, and the tortured mouth settle down into a content that was ineffable.

"Miss Storkey was here yesterday," went on the child, brokenly, "and the band was a-playin' beautiful, and—and she knocked it over accidental—and now its broke!"

Then the storm of grief burst out afresh.

Miss Storkey again! The poor district-visitor seemed fated to make herself unpleasant.

"Jarvy!" I spoke after a long pause. "I, too, have a 'band'—a valuable musical box, which belonged to my dear mother. It has never played since she went home to heaven; but, somehow, I think she'd like me to lend it to you—for a little while." Who knew better than I that the loan would be but a short one? The wings of Jarvy's little white soul were pushing for flight; and something told me that the child knew it as well as I.

"Thanky'e, miss; I'll be very keerful of the band—while I has it," said he soberly enough, but his face all alight with joy; by-and-bye, when mother's musical-box trilled out its melodies, made me ashamed of my fleeting grudge at lending it.

It had come; the hasty summons that I knew must, sooner or later, reach me. Little Jarvy was dying! With flying feet I arrived at the cottage, but the angels had already been and gone. There was the unmistakeable hush that their presence leaves as I entered, but the stillness was broken by a strain of thrilling sweetness:—

> "Angels ever bright and fair,
> Take, oh take me to thy care!"

Was the little marble form that lay so still on Jarvy's bed, with fair white lids softly closing over tired blue eyes, and hands meekly folded, listening to the melody?

"You'll pardon it, miss, but it will keep on a-chimin'!" It was Gran's tearless voice, speaking tremulously. "The dear lamb, he called out jest as he was goin', 'make the band play!' Then a great light sprang into his face, and he cried out, 'They're comin', all the angels, in white crowds!' And then—he went with them."

There was no doubt of *that*. Maimed little suffering Jarvy had gone with the white crowds to listen to other and far sweeter music than that of the "band."

### Appointments.

Lytham Cottage Hospital.—Miss Isabel Firth has been unanimously elected Matron of this hospital and convalescent home. Miss Firth is the daughter of the late Mr. G. W. W. Firth, F.R.C.S., and J.P., of Norwich. Miss Firth trained at Addenbrooke's under the late Miss Fisher, and has worked at Pendlebury and the London Fever Hospital, and has for two years been Matron of the Brentwood Cottage Hospital. Her testimonials are excellent.

Taunton Sanitary Hospital.—Miss Emily Stendell has been appointed Matron of this hospital. She trained at Leeds Infirmary, and also acted as Sister there for nearly three years. Miss Stendell has lately been working at the Bristol Children's Hospital, where she has won golden opinions.

Croydon Infirmary.—Miss Julian, now Matron of St. Saviour's Infirmary, has been appointed to Croydon.

### Amusements and Relaxation.

### SPECIAL NOTICE TO CORRESPONDENTS.

First quarterly word competition commenced January 3rd, 1891; ends March 28th, 1891.

Competitors can enter for *all* quarterly competitions, but no competitor can take more than *one first prize* or *two* prizes of any kind during the year.

The word for dissection for this, the TWELFTH week of the quarter, being "BLIZZARD,"

| Names. | March 11th. | Totals. | Names. | March 11th. | Totals. |
|---|---|---|---|---|---|
| Reynard | — | 77 | Woodbine | — | 25 |
| Reldas | 14 | 534 | Madame B. | — | 25 |
| Tinie | — | 30 | Shakespeare | — | 59 |
| Patience | — | 76 | Smyrna | — | 259 |
| Jenny Wren | 13 | 477 | Southwood | — | 102 |
| Agamemnon | 14 | 527 | Gipsy Queen | — | 21 |
| Wyameris | — | 391 | Snowball | — | 19 |
| E. C. | 14 | 524 | Rita | — | 88 |
| Ecila | — | 283 | Mortal | — | 16 |
| Hope | 14 | 531 | Nurse Annie | — | 15 |
| M. W. | 14 | 520 | Carmen | — | 11 |
| Qu'appelle | 14 | 516 | Grannie | — | 45 |
| Nil Desperandum | 14 | 524 | Amie | — | 30 |
| Lady Betty | 14 | 487 | M. R. | — | 25 |
| H. A. S. | — | | Primrose | — | 24 |
| Sister Jack | — | 62 | Nurse J. S. | 12 | 292 |
| Crystal | — | 203 | B. A. C. | 14 | 223 |
| | | | Theta | 13 | 118 |

**Notice to Correspondents.**

N.B.—*On account of the Easter Holidays all word dissections must reach the office not later than the first post on Wednesday morning next.*

Second Quarterly Word Competition commences April 4th; ends June 27th, 1891.

## Figures and Tables

**Figure f1:**